# Performance Analysis of Millimeter-Wave UAV Swarm Networks under Blockage Effects

**DOI:** 10.3390/s20164593

**Published:** 2020-08-16

**Authors:** Haejoon Jung, In-Ho Lee

**Affiliations:** 1 Department of Information and Telecommunication Engineering, Incheon National University, Incheon 22012, Korea; haejoonjung@inu.ac.kr; 2 School of Electronic and Electrical Engineering, Hankyong National University, Anseong 17579, Korea

**Keywords:** unmanned aerial vehicles (UAVs), millimeter wave (mmWave), UAV swarm network

## Abstract

Due to their high mobility, unmanned aerial vehicles (UAVs) can offer better connectivity by complement or replace with the existing terrestrial base stations (BSs) in the mobile cellular networks. In particular, introducing UAV and millimeter wave (mmWave) technologies can better support the future wireless networks with requirements of high data rate, low latency, and seamless connectivity. However, it is widely known that mmWave signals are susceptible to blockages because of their poor diffraction. In this context, we consider macro-diversity achieved by the multiple UAV BSs, which are randomly distributed in a spherical swarm. Using the widely used channel model incorporated with the distance-based random blockage effects, which is proposed based on stochastic geometry and random shape theory, we investigate the outage performance of the mmWave UAV swarm network. Further, based on our analysis, we show how to minimize the outage rate by adjusting various system parameters such as the size of the UAV swarm relative to the distance to the receiver.

## 1. Introduction

Unmanned aerial vehicle (UAV)-assisted communications have attracted significant research attention as a promising technology to support emergent and ad hoc communications for the ground and low-altitude users by deploying UAVs [1,2,3,4]. Serving as aerial base stations (BSs) in various applications, UAVs can be used to achieve higher capacity, wider coverage, higher energy efficiency, and higher secrecy rate [5,6,7,8,9,10]. For this reason, UAV BSs are more cost-effective compared to the conventional terrestrial BSs, benefiting from their capabilities to dynamically adjust their positions and altitudes [11]. In particular, deploying as a group, UAV swarm can be used to retrieve a line-of-sight (LoS) path to users, which can provide better connectivity to users compared to terrestrial BSs, because terrestrial BSs frequently suffer from physical obstacles such as buildings and mountains.

When it comes to millimeter-wave (mmWave) communication, this blockage by the obstacles become more critical compared to sub-6 GHz frequency bands. While mmWave bands can provide higher data rate for fifth-generation (5G) and beyond 5G (B5G) cellular networks with its more abundant bandwidth from 30 to 300 GHz, mmWave signals undergo severe attenuation due to significant path-loss and absorption. To overcome this issue, beamforming techniques based on large-scale antenna arrays have been employed, which compensate for the higher attenuation compared to the sub-6 GHz bands. For this reason, mmWave networks typically rely on highly directional beams, which makes them susceptible to blockage, because of the short wavelengths of the mmWave and the corresponding poor diffraction [12]. As a result, the mmWave channel is almost bimodal, which is subject to the presence or absence of the LoS path [13]. Motivated by this characteristic of the mmWave channels, the authors of [14,15,16] propose a channel model incorporated with the blockage effects in aid of random shape theory in [17]. In the proposed model, the probability that the blockage-free LoS path exists is an exponentially decreasing function of the Euclidean distance between the transmitter and the receiver. This model has been widely used to analyze the mmWave network performance as in [18,19,20,21].

Considering the larger available frequency band at mmWave to resolve rapidly increasing capacity demand in the future wireless systems, the employment of mmWave technology is inevitable in the UAV-assisted networks. In fact, the combination of UAV and mmWave techniques can be a key enabler of the 5G and B5G networks by efficient spectrum management protocols [22]. As noted in [23,24,25,26], when a UAV swarm as a collaborative team is enabled with mmWave technology, it can provide emergency communications for natural disasters and meet intense service requests in crowded areas, which the conventional terrestrial BSs cannot fully function. Furthermore, integration of UAV and mmWave can be a promising solution in the heterogeneous networks by multi-connectivity approach [27,28].

In this context, we consider a mmWave-enabled UAV network, where a UAV swarm provide connectivity to a far-distance user in the presence of random obstacles (i.e., blockages). Following the blockage model in [14], we investigate the probability to construct a LoS link between the UAV BSs in the swarm and the receiver. To be specific, extending from the two-dimensional network model assuming terrestrial BSs in [14,18,19], we analyze the outage probability of the network with various system parameters such as the density of the UAV BSs, blockage constant, the size of the UAV swarm, and the distance to the receiver. The main contributions of this paper are summarized as follows:

- We present the statistics of the distance between an arbitrary UAV BS in a sphere-shaped swarm to the user. To be specific, the probability density function (PDF) of the distance is derived, and its approximate version to obtain the first and second moments in closed forms is also provided.
- Assuming random blockages between the UAV swarm and the receiver, we derive a closed-form expression of the outage rate, which is defined by the likelihood to have no LoS path.
- Using the derived outage rate, we present how it changes with various system parameters. In particular, we show that there exists an optimal value of the size of the UAV swarm to minimize the outage probability. Further, the optimal size of the spherical UAV swarm is derived in a closed-form.
- We present both simulation and theoretical results to show the impact of various system parameters. In addition, by comparing the two results, we validate our analysis.

The rest of this work is organized as follows. In Section 2, we present prior studies related to this work. In Section 3, we introduce the system model including network topology. In Section 4, we study the statistics of the distance between the UAV BSs and the receiver, since the blockage effect is subject to the distance of the link. In Section 5, we analyze the outage probability and show how to optimize the outage performance by adjusting different system parameters. In Section 6, simulation and numerical results are presented. We conclude our paper in Section 7.

## 2. Related Work

To cope with the rapidly increasing data traffic and number of devices in 5G and B5G [29], network densification is an effective means to enhance capacity and coverage [30]. In other words, the future mobile networks are expected to deploy more BSs (typically small cell BSs), which is referred to as ultra-dense networks (UDNs), as a solution to satisfy the skyrocketing communication demands of diverse types of user equipments (UEs) [31]. In the UDNs, the increased density of access points (APs) in various types enables to achieve higher spatial reuse of wireless resources [32,33,34]. Further, through short inter-site distances and low interference levels, the spatial reuse of the UDNs has two major advantages: enhanced network capacity and improved link quality [33].

The integration of UDNs and mmWave technology can boost both energy efficiency and spectral efficiency [35]. Since the attenuation of the mmWave frequency band is significantly higher compared to the sub-6GHz, the corresponding interference levels at mmWave are vastly lower. In addition, the abundant available bandwidth of the mmWave band can provide considerably higher data rates [36]. As a result, such physical properties of mmWave make it play a key role in increasing the spectral efficiency and energy efficiency of the UDNs. Moreover, mmWave can be used to construct wireless backhaul, because the dense deployment of BSs will make conventional wired backhaul challenging [18,19,37,38].

However, the traditional terrestrial infrastructure with fixed location is not appropriate in urban networks with high mobility. In this context, UAVs have distinctive advantages to employ as flying BSs with capabilities to provide connectivity for users in overloaded areas [39]. However, because UAVs can cause strong interference due to the strong LoS component, which is the inherent characteristic of air-to-ground (A2G) channels, UAV-aided UDNs require techniques to use highly directional beams at mmWave, which can also support multiple users simultaneously by deploying multiple UAVs in the form of a UAV swarm [40,41,42,43,44]. For this reason, in this paper, we integrate the mmWave communication with multiple UAV BSs and consider how to operate them to reduce outage probabilities through spatial diversity [45,46,47]. In particular, we note that this is the first study to identify how to optimize the mmWave UAV swarm networks using various system parameters such as the density of UAV BSs and the size of the UAV swarm for a given degree of the blockage effect.

## 3. System Model

**Figure 1:**
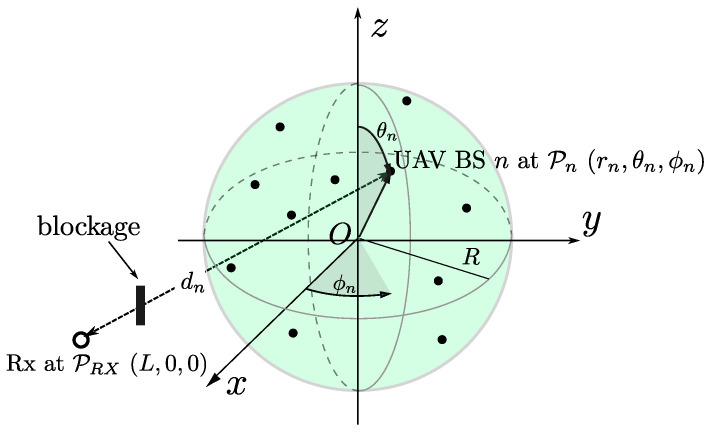
Illustration of millimeter wave (mmWave) fifth-generation (5G) cellular network.

Figure 1 illustrates our system model, in which the black dots and the white-filled circle represent UAV BSs and the desired receiver (Rx). We assume that the number of UAV BSs is *N* UAVs, while there exists a single receiver, which is located at (L,0,0) in the spherical coordinates with the origin labeled with *O*. As indicated by the sphere in Figure 1, we assume the UAV BSs are uniformly distributed over the sphere with radius of *R* with intensity λ according to homogeneous Poisson point process (PPP). In other words, the average number of UAV BSs per a unit volume is λ. Therefore, the number of UAV BSs *N*, which corresponds to the number of the dots in Figure 1, is a Poisson random variable, the probability mass function of which is given by

(1) $$\begin{array}{c} P_{N}\left(n\right)=\frac{{\left(\frac{4}{3}\lambda \pi R^{3}\right)}^{n}}{n!}\exp\left(-\frac{4}{3}\lambda \pi R^{3}\right), \end{array}$$

where *N* is a non-negative integer (i.e., N≥0). Hence, $P_{N}\left(n\right)$ corresponds to the probability that *n* UAV BSs exist in the sphere. In addition, the average of number of the UAV BSs is

(2) $$\begin{array}{c} 𝔼\left[N\right]=\frac{4}{3}\lambda \pi R^{3}. \end{array}$$

On the other hand, there is a single mmWave BS, which does not belong to the legacy network and is separated by a length *L* from the center of the sphere 𝓢.

As labeled with UAV BS *n* in Figure 1, the location of the *n*-th UAV BS is denoted by $\left(r_{n},\theta_{n},\phi_{n}\right)$. Further, the links between the UAV BSs and the receiver suffer from random blockage effects. For example, in Figure 1, the LoS path between the *n*-th UAV BS and the receiver is blocked. To consider the 3D uniform distribution, we define the following function

(3) $$\begin{array}{c} V\left(r,\theta ,\phi \right)=\int_{0}^{r}\int_{0}^{\theta }\int_{0}^{\phi }r^{2}\sin\theta d\phi d\theta dr. \end{array}$$

Then, the volume of the sphere with the radius *R* shown in Figure 1 is $V_{s}=V\left(R,\pi ,2\pi \right)$. Therefore, for the uniform distribution within $V_{s}$, the marginal probability density functions (PDFs) of $r_{n}$, $\theta_{n}$, and $\phi_{n}$ are given by

(4) $$\begin{array}{cc} \; & f_{r_{n}}\left(r\right)=\frac{\partial V}{\partial r}\frac{1}{V_{s}}=\frac{3r^{2}}{R^{3}}, \end{array}$$

(5) $$\begin{array}{cc} \; & f_{\phi_{n}}\left(\phi \right)=\frac{\partial V}{\partial \phi }\frac{1}{V_{s}}=\frac{1}{2\pi }, \end{array}$$

(6) $$\begin{array}{cc} \; & f_{\theta_{n}}\left(\theta \right)=\frac{\partial V}{\partial \theta }\frac{1}{V_{s}}=\frac{\sin\theta }{2}, \end{array}$$

respectively. Because the three random variables are independent, their joint PDF is given by

(7) $$\begin{array}{c} f_{r_{n},\theta_{n},\phi_{n}}\left(r,\theta ,\phi \right)=\frac{3r^{2}\sin\theta }{4\pi R^{3}}, \end{array}$$

where 0≤r≤R, 0≤θ≤π, and −π≤ϕ<π. If converting the node location into the Cartesian coordinates, the marginal PDFs of $x_{n}$, $y_{n}$, and $z_{n}$ are expressed as

(8) $$\begin{array}{cc} x_{n} & =r_{n}\sin\theta_{n}\cos\phi_{n}, \end{array}$$

(9) $$\begin{array}{cc} y_{n} & =r_{n}\sin\theta_{n}\sin\phi_{n}, \end{array}$$

(10) $$\begin{array}{cc} z_{n} & =r_{n}\cos\theta_{n}. \end{array}$$

Further, their joint PDF is given by

(11) $$f_{x_{n},y_{n},z_{n}}\left(x,y,z\right)=\frac{3}{4\pi R^{3}},$$

where −R≤x, *y*, z≤R. The separation between the center of the sphere and the receiver is defined as L=ρR, where ρ is the ratio between the separation *L* to the radius of the sphere *R*, and ρ>1. Thus, without loss of generality based on symmetry, the location of the received is (L,0,0) in the Cartesian coordinates. Hence, the Euclidean distance between the *n*-th UAV BS, located at $\left(x_{n},y_{n},z_{n}\right)$ according to the Cartesian coordinates, and the receiver is given by

(12) $$\begin{array}{cc} d_{n} & ={\left[{\left(L-r_{n}\sin\theta_{n}\cos\phi_{n}\right)}^{2}+r_{n}^{2}\sin^{2}\theta_{n}\sin^{2}\phi_{n}+r_{n}^{2}\cos^{2}\theta_{n}\right]}^{1/2}\\ \; & ={\left[L^{2}+r_{n}^{2}-2Lr_{n}\sin\theta_{n}\cos\phi_{n}\right]}^{1/2}. \end{array}$$

Hence, the PDF of $d_{n}$ can be derived by the change of variable from the joint PDF in (7).

## 4. Distance Distribution and Path Loss Statistics

**Figure 2:**
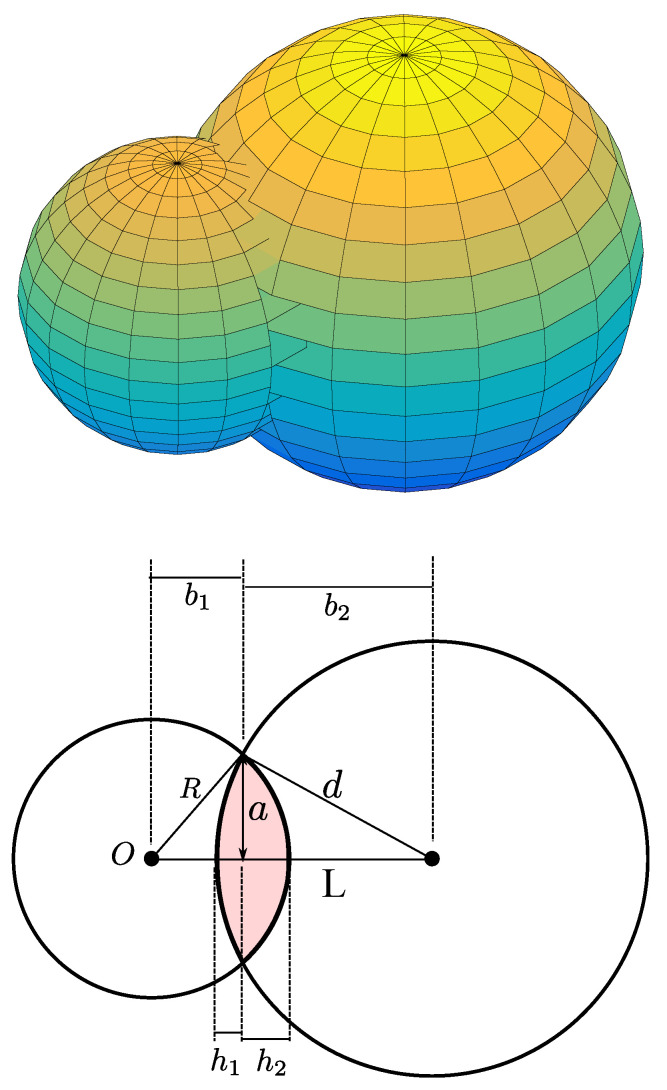
The geometry underlying the probability distribution of $d_{n}$.

In this section, we derive the PDF of the distance $d_{n}$ between the *n*-th UAV BS and the receiver to investigate the performance of the cooperative transmissions using the multiple UAV BSs. Figure 2 illustrates an example geometry to derive the PDF of $d_{n}$. As shown in Figure 2, we assume that the origin *O* is the center of the sphere, within which the transmitting UAV BSs are uniformly distributed. In addition, without loss of generality, the receiver is located at (x,y,z)=(L,0,0). The right sphere is centered at the receiver with radius of $d_{n}$, as shown in the cross-section. In addition, the shaded area is the cross-sectional area of the volume of the 3D lens common to the two spheres, which is denoted by $V_{l}$. Hence, the cumulative distribution function (CDF) of $d_{n}=d$, $F_{d_{n}}\left(d\right)$ can be derived as the ratio of $V_{l}$ to $V_{s}=\frac{4}{3}\pi R^{3}$, based on the definition of the CDF as $F_{d_{n}}\left(d\right)=\mathrm{Pr}\left[d_{n}\leq d\right]$.

The equations of the two spheres in Figure 2 are

(13) $$\begin{array}{c} x^{2}+y^{2}+z^{2}=R^{2}, \end{array}$$

(14) $$\begin{array}{c} {\left(x-L\right)}^{2}+y^{2}+z^{2}=d^{2}, \end{array}$$

respectively. Combining the two equations, we obtain

(15) $$\begin{array}{c} {\left(x-L\right)}^{2}+\left(R^{2}-x^{2}\right)=d^{2}, \end{array}$$

which gives $x=\frac{L^{2}+R^{2}-d^{2}}{2L}$. Therefore, the intersection of the two spheres, which is a curve lying in a plane parallel to the z-plane, is expressed as

(16) $$\begin{array}{c} y^{2}+z^{2}=a^{2}, \end{array}$$

where $a=\frac{1}{2L}{\left[4L^{2}R^{2}-{\left(L^{2}+R^{2}-d^{2}\right)}^{2}\right]}^{1/2}$. Hence, $V_{l}$ can be given by the sum of two spherical caps with the bases of

(17) $$\begin{array}{c} b_{1}=x=\frac{L^{2}+R^{2}-d^{2}}{2L}, \end{array}$$

(18) $$\begin{array}{c} b_{2}=L-b_{1}=\frac{L^{2}-R^{2}+d^{2}}{2L}, \end{array}$$

respectively. Correspondingly, the heights of the two spherical caps, which are denoted by $h_{1}$ and $h_{2}$ in Figure 2 are given by

(19) $$\begin{array}{c} h_{1}=d-b_{2}=\frac{2Ld-L^{2}-d^{2}+R^{2}}{2L}, \end{array}$$

(20) $$\begin{array}{c} h_{2}=R-b_{1}=\frac{2LR-L^{2}+d^{2}-R^{2}}{2L}, \end{array}$$

respectively. The volume of a spherical cap with height of *h* for a sphere of radius *r*, which is shown in Figure 3, is expressed as

(21) $$\begin{array}{c} V_{c}\left(r,h\right)=\frac{1}{3}\pi h^{2}\left(3r^{2}-h\right). \end{array}$$

Therefore, the sum of the volumes of the two spherical caps in Figure 2 is given by

(22) $$\begin{array}{cc} \; & V_{l}=V_{c}\left(R,h_{2}\right)+V_{c}\left(d,h_{1}\right). \end{array}$$

**Figure 4:**
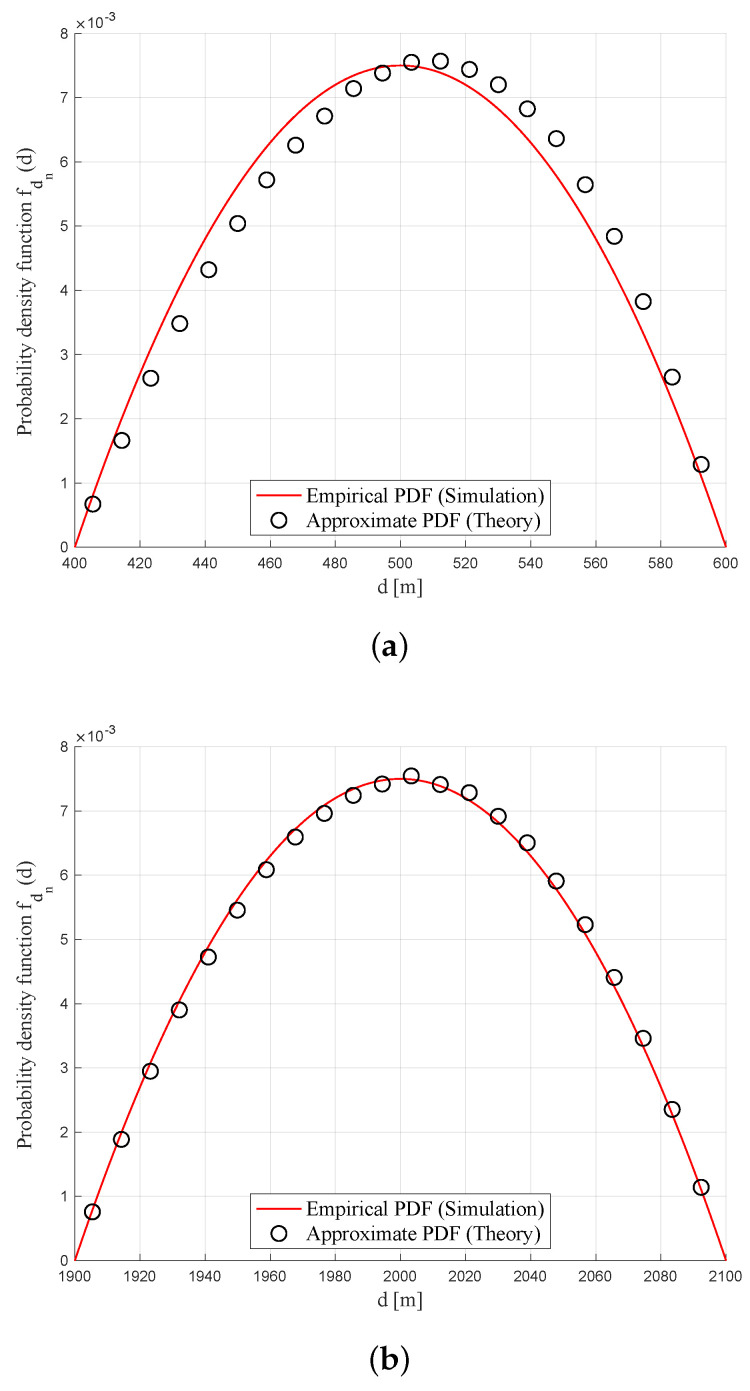
Comparison of the approximate probability density function (PDF) of $d_{n}$ and its empirical PDF based on simulation for R=100m. (**a**) L=300 m (ρ=3); (**b**) L=2 km (ρ=20).

As a result, the CDF of $d_{n}$ is given by

(23) $$\begin{array}{cc} F_{d_{n}}\left(d\right)= & \frac{V_{l}}{V_{s}}\\ = & \frac{1}{4R^{3}}[\left(\frac{L^{2}+d^{2}-R^{2}}{2L}+3d^{2}-d\right){\left(\frac{R^{2}-L^{2}-d^{2}}{2L}+d\right)}^{2}\\ \; & +{\left(R-\frac{L^{2}-d^{2}+R^{2}}{2L}\right)}^{2}\left(\frac{L^{2}-d^{2}+R^{2}}{2L}+3R^{2}-R\right)]. \end{array}$$

where L−R≤d≤L+R. Thus, its PDF is the derivative of $F_{d_{n}}\left(d\right)$. However, this exact PDF does not give the mean and variance in closed-form expressions, which makes the outage performance analysis challenging. For this reason, we propose an approximate PDF, which is accurate enough for the far-field scenario with L≫R. The key idea is to approximate the surface of the spherical cap, which corresponds to the differential volume element as indicated as the blue surface in Figure 3, by the cross-section disk area indicated by the green area in Figure 3. Based on this simplification, the approximate PDF is given by

(24) $$\begin{array}{cc} f_{d_{n}}^{*}\left(d\right)\; & =\frac{3\left(R^{2}-{\left(L-d\right)}^{2}\right)}{4R^{3}}, \end{array}$$

where L−R<d<L+R. Figure 4a,b show the two PDFs for L=200 m, 400 m when R=100, respectively. In each graph, the solid and dotted lines indicate the exact and approximate PDFs, which corresponds to the derivative of the CDF in (23) and the PDF in (24), respectively. As shown in the figures, $f_{d_{k}}\left(x\right)\approx f_{d_{k}}^{*}\left(x\right)$ in the entire domain L−R≤x≤L+R.

Using the approximate PDF $f_{d_{n}}^{*}\left(d\right)$ in (24), the average and variance of $d_{n}$ are obtained as

(25) $$\begin{array}{c} 𝔼\left\{d_{n}\right\}=\int_{L-R}^{L+R}xf_{d_{k}}^{*}\left(x\right)dx=L, \end{array}$$

(26) $$\begin{array}{cc} 𝕍𝔸\mathbb{R}\{d_{n}\} & =𝔼\left[d_{n}^{2}\right]-{\left(𝔼\left[d_{n}\right]\right)}^{2}=\int_{L-R}^{L+R}x^{2}f_{d_{k}}^{*}\left(x\right)dx-L^{2}\\ \; & =L^{2}+\frac{R^{2}}{5}-L^{2}=\frac{R^{2}}{5}. \end{array}$$

## 5. Outage Probability Analysis

In this section, we investigate the outage probability, which is defined as the probability that all UAV BSs in the spherical volume 𝓢 cannot reach the receiver through the LoS path because of the all LoS paths are blocked by obstacles. To analyze this outage event, following the framework in [14,18,19], we define a Bernoulli random variable $U_{n}$, where one represents that the *n*-th link between UAV BS *n* and the receiver is reliable (i.e., the LoS path exists). On the other hand, zero corresponds to the outage event of the *n*-th link (i.e., the LoS path is blocked). The probability of this Bernoulli random variable is defined as

(27) $$U_{n}=\left\{\begin{array}{cc} 1, & \mathrm{with}\;\mathrm{probability}\;\exp\left(-\beta d_{n}\right),\\ 0, & \mathrm{with}\;\mathrm{probability}\;1-\exp\left(-\beta d_{n}\right), \end{array}\right.$$

where n={1,2,…,N}. As in [14,18], assuming impenetrable blockage, an outage event of the UAV network is defined as the case that all of the $U_{n}$’s are zeros for all 1≤n≤N. That is, in case that there exists at least one UAV BS *n* with $U_{n}=1$ for all *n*, the receiver can communicate with the core network. Hence, the outage of the network $P_{out}$ can be defined as

(28) $$\begin{array}{cc} P_{out} & =\sum_{k=1}^{\infty }\mathrm{Pr}\left[U_{n}=0\;\mathrm{for}\;\mathrm{all}\;1\leq n\leq k\right]\cdot \mathrm{Pr}\left[N=k\right]+\mathrm{Pr}\left[N=0\right], \end{array}$$

where 1≤n≤k.

As in [14,19], we assume the Bernoulli random variables $U_{n}$ with different *n* in (28) are independent. Under the assumption of independent blockage events, the outage probability in (28) is expressed as

(29) $$P_{out}=\sum_{k=1}^{\infty }\left(\prod_{n=1}^{k}𝔼\left[1-\exp\left(-\beta d_{n}\right)\right]\right)\mathrm{Pr}\left[N=k\right]+\mathrm{Pr}\left[N=0\right],$$

where $d_{n}$ is the distance between the *n*-th UAV BS and the receiver following the approximate PDF in (24). In addition, the expectation term $𝔼[1-\exp\left(-\beta d_{n}\right)]$ can be obtained as

(30) $$\begin{array}{cc} 𝔼[1-\exp\left(-\beta d_{n}\right)] & =1-𝔼[\exp\left(-\beta d_{n}\right)]\\ \; & =1-\int_{L-R}^{L+R}e^{-\beta x}f_{d_{n}}\left(x\right)dx\\ \; & \overset{\left(a\right)}{\approx }1-\int_{L-R}^{L+R}e^{-\beta x}f_{d_{n}}^{*}\left(x\right)dx\\ \; & =1-\int_{L-R}^{L+R}e^{-\beta x}\frac{3\left(R^{2}-{\left(L-x\right)}^{2}\right)}{4R^{3}}dx\\ \; & \overset{\left(b\right)}{\approx }1-\left(1+\frac{\beta^{2}𝕍𝔸\mathbb{R}\left[d_{n}\right]}{2}\right)\exp\left(-\beta 𝔼\left[d_{n}\right]\right)\\ \; & =1-\left(1+\frac{\beta^{2}R^{2}}{10}\right)\exp\left(-\beta L\right), \end{array}$$

where (a) follows from the approximation of the PDF given in (24). In addition, (b) follows from Taylor expansion. Consequently, the outage rate in (29) can be derived in a closed-form expression as

(31) $$\begin{array}{cc} P_{out} & =\sum_{k=0}^{\infty }{\left(𝔼[1-\exp\left(-\beta d_{n}\right)]\right)}^{k}\cdot \mathrm{Pr}\left[K=k\right]\\ \; & \overset{\left(a\right)}{\approx }\sum_{k=0}^{\infty }{\left(1-\left(1+\frac{\beta^{2}R^{2}}{10}\right)\exp\left(-\beta L\right)\right)}^{k}\cdot \frac{{\left(\frac{4}{3}\lambda \pi R^{3}\right)}^{k}}{k!}e^{-\frac{4}{3}\lambda \pi R^{3}}\\ \; & =\exp\left(-\frac{4}{3}\lambda \pi R^{3}\left(1+\frac{\beta^{2}R^{2}}{10}\right)\exp\left(-\beta L\right)\right), \end{array}$$

where (a) follows from the approximation in (30).

Based on this derived outage probability, we can identify the impacts of various system parameters on the outage performance of the system, which provide insights into the mmWave UAV swarm network design and implementation under certain constraints. In other words, it is critical to minimize the outage probability, which is subject to the channel characteristics quantified by β and the distance to the receiver *L*, by adjusting the UAV density λ and the size of the swarm *R*. For this reason, we provide the following nine properties to better understand how the outage rate behaves as the various system parameters vary, which ultimately help optimizing the overall system performance. We note that the properties given in this section will be be validated by comparing with simulation results in the next section.

**Property 1**. *(Impact of λ)—The outage rate in * (31) *is a decreasing function of λ, when the other parameters R, λ, β, and L are fixed. In other words, when λ, β, and L=ρR are all positive, we have the limiting values of the outage probability as*

(32) $$\begin{array}{c} \lim_{\lambda \to \infty }P_{out}=0,\\ \lim_{\lambda \to 0}P_{out}=1. \end{array}$$

**Property 2**. *(Impact of β)—As the blockage parameter β→∞, which means that the probability of blockage increases, the outage probability becomes*

(33) $$\begin{array}{c} \lim_{\beta \to \infty }P_{out}=1. \end{array}$$

*On the other hand, when β→0, we have*

(34) $$\begin{array}{c} \lim_{\beta \to 0}P_{out}=\exp\left(\frac{4}{3}\lambda \pi R^{3}\right), \end{array}$$

*which corresponds to the probability that there is no UAV BS in the given spherical volume.*

**Property 3**. *(Optimal β)—Based on the two limiting values in Property 2, we can check if there exists an optimal value of β that maximizes $P_{out}$ in * (31)*. In case that there exists an optimum, we can find it by*

(35) $$\begin{array}{c} \frac{\partial P_{out}\left(R,\beta ,L=\rho R\right)}{\partial \beta }=0, \end{array}$$

*which corresponds to*

(36) $$\begin{array}{c} \beta^{*}=\frac{R\pm \sqrt{R^{2}-10\rho^{2}R^{2}}}{\rho R^{2}}. \end{array}$$

However, because ρ>1, $\beta^{*}$ satisfying  (36) *cannot be found. Therefore, we can conclude that $P_{out}$ is minimized, when β=0.*

**Property 4**. *(Impact of R)—For λ>0, β>0, and ρ>1 (i.e., L>R), when R→0, the outage probability becomes*

(37) $$\begin{array}{c} \lim_{R\to 0}P_{out}=1. \end{array}$$

*On the other extreme, as R→∞, we get*

(38) $$\begin{array}{c} \lim_{R\to \infty }P_{out}=1, \end{array}$$

*because the exponential term $e^{-\beta L}=e^{-\beta \rho R}$ is dominant and it goes zero as R→∞.*

**Property 5**. *(Optimal Value of R)—For given λ>0, β>0, and ρ>1, the outage rate in * (31) *is minimized, when the radius R is equal to $R_{opt}$ in * (40)*, by taking the derivative of * (31) *with respect to R and solving*

(39) $$\frac{\partial P_{out}\left(R,\beta ,L=\rho R\right)}{\partial R}=0.$$

We note that $R_{opt}^{*}$ in  (40) is a unique solution that also suffices the second derivative condition for the minimum value (i.e., $\frac{\partial^{2}P_{out}^{*}}{\partial^{2}R}>0$).

(40) $$\begin{array}{cc} R_{opt} & =\frac{\sqrt[{3}]{5}\sqrt[{3}]{3\sqrt{6}\sqrt{20\beta^{12}\rho^{6}-26\beta^{12}\rho^{4}+75\beta^{12}\rho^{2}}+36\beta^{6}\rho^{2}+25\beta^{6}}}{3\beta^{3}\rho }\\ \; & -\frac{30\beta^{4}\rho^{2}-25\beta^{4}}{3\sqrt[{3}]{5}\beta^{3}\rho \sqrt[{3}]{3\sqrt{6}\sqrt{20\beta^{12}\rho^{6}-26\beta^{12}\rho^{4}+75\beta^{12}\rho^{2}}+36\beta^{6}\rho^{2}+25\beta^{6}}}+\frac{5}{3\beta \rho }. \end{array}$$

**Property 6**. *(Impact of $\rho =\frac{L}{R}$)—When the other parameters λ, β, and R are given, as the ratio $\rho =\frac{L}{R}$ increases, the outage probability in* (31) *decreases. This can be explained by the growing separation between the center of the spherical volume, where UAV BSs are distributed, and the receiver.*

**Property 7**. *($R_{opt}$ and λ)—As indicated by * (40)*, $R_{opt}$ is not a function of λ. In other words, varying λ does not change the optimal value of the radius of the spherical volume.*

**Property 8**. *($R_{opt}$ and β)—The radius $R_{opt}$ in* (40) *is a decreasing function of β.*

**Property 9**. *($R_{opt}$ and $\rho =\frac{L}{R}$)—It is noted that the optimal value of the radius $R_{opt}$ in * (40) *is a decreasing function of ρ.*

## 6. Numerical Results

In this section, we present simulation results, comparing with numerical results based on the analysis in the previous section. Using both results, we investigate how the outage probability changes under the changes in various system parameters.

### 6.1. Impact of R on $P_{out}$

**Figure 5:**
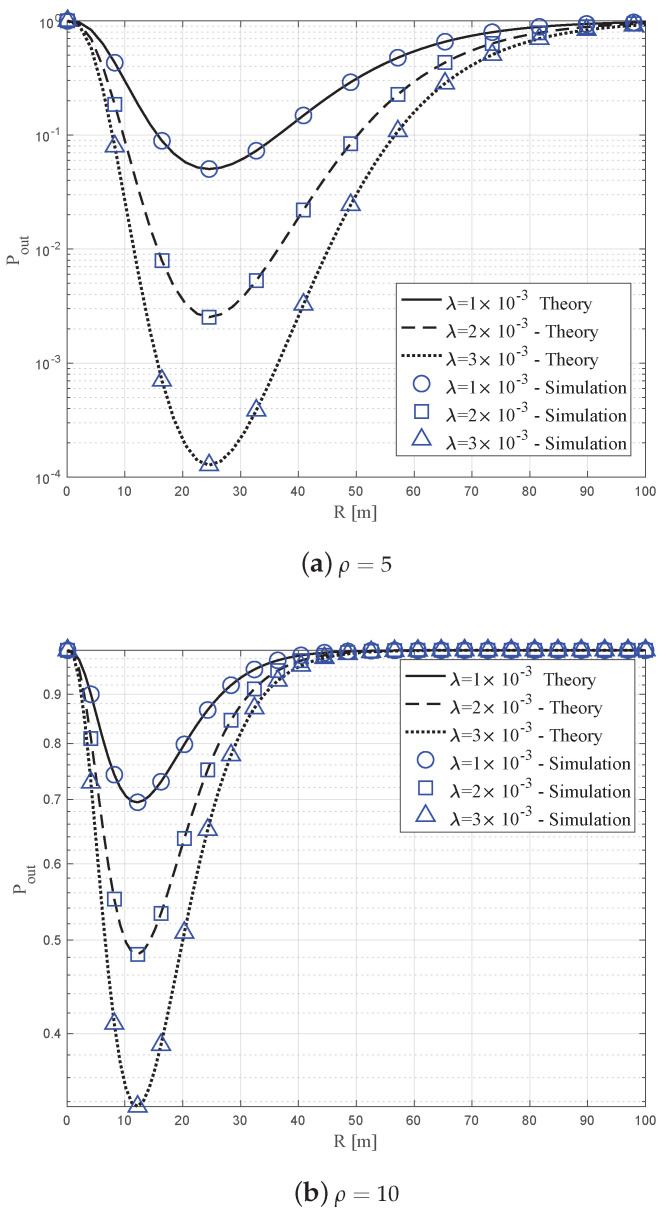
Outage probability versus radius *R* with β=0.025.

Figure 5 shows the outage probability $P_{out}$ with varying radius of the spherical volume *R*, when β=0.025. Figure 5a,b correspond to the different ratios $\rho =\frac{L}{R}$ of 5 and 10, respectively. In each figure, the horizontal axis indicates the radius *R*, while the vertical axis corresponds to the outage rate. Further, the solid, dashed, and dotted lines represent numerical results derived as (31) for λ={0.001,0.002,0.003}, respectively. On the other hand, the circle, square, and triangle markers respectively represent the exact outage probabilities obtained by simulation with $10^{7}$ random topology realizations of the UAVs and blockages.

In the figures, we first observed that the simulation and theoretical results showed good correlation, which validated our analysis. In addition, as stated in Property 1, at the same *R*, the outage rate with higher λ was smaller compared to that with lower λ. This is because it was likely to have more number of UAV BSs, which provided diversity, as λ increased. Further, the graphs show that the outage probability was not a monotonically increasing nor decreasing function of the radius *R*, as presented in Property 4. Instead, for given λ and β, it was a convex function, where there existed an optimal value of *R* that minimized the outage probability. In both Figure 5a,b, interestingly, the optimal value of *R* did not change with different λ, which is in line with our finding in Property 7 in the previous section.

### 6.2. Impacts of ρ and β on $P_{out}$

**Figure 6:**
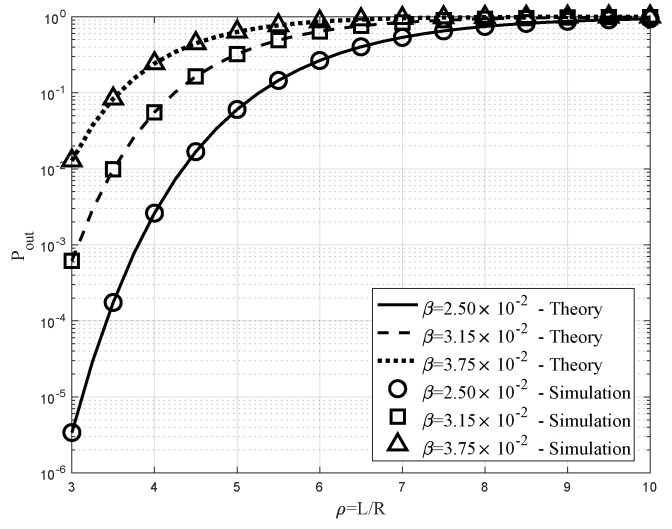
Outage rate versus ρ=L/R with $\lambda =10^{-3}$R=30m, and $\beta =\{2.5\times 10^{-2},\;3.15\times 10^{-2},\;3.75\times 10^{-2}\}$.

Figure 6 illustrates how the outage probability $P_{out}$, which is indicated by the y-axis, varied under the change in ρ=L/R, which is represented by the x-axis. We chose fixed parameters of R=30 m and λ=0.001, while the three different values of $\beta =\{2.5\times 10^{-2},3.15\times 10^{-2},3.75\times 10^{-2}\}$ were used, which correspond to the solid, dashed, and dotted lines in case of the theoretical results, respectively. On the other hand, the circle, square, and triangle markers indicate to the corresponding simulation results. In this figure, we also observed good correlation of the simulation and theoretical results, which again validated the analysis in the previous section. As provided in Property 9, all of the three graphs and markers showed the same increasing trend of the outage rate $P_{out}$ as the ratio ρ increased, because the likelihood of a blockage even on each link grew with increasing ρ. In addition, as stated in Property 3, we observed that the outage with the lowest β was the smallest compared to the other curves at the same ρ. This can also be explained by the increased probability of the blockage by the increase in the blockage constant β.

### 6.3. Impacts of ρ and λ on $P_{out}$

**Figure 7:**
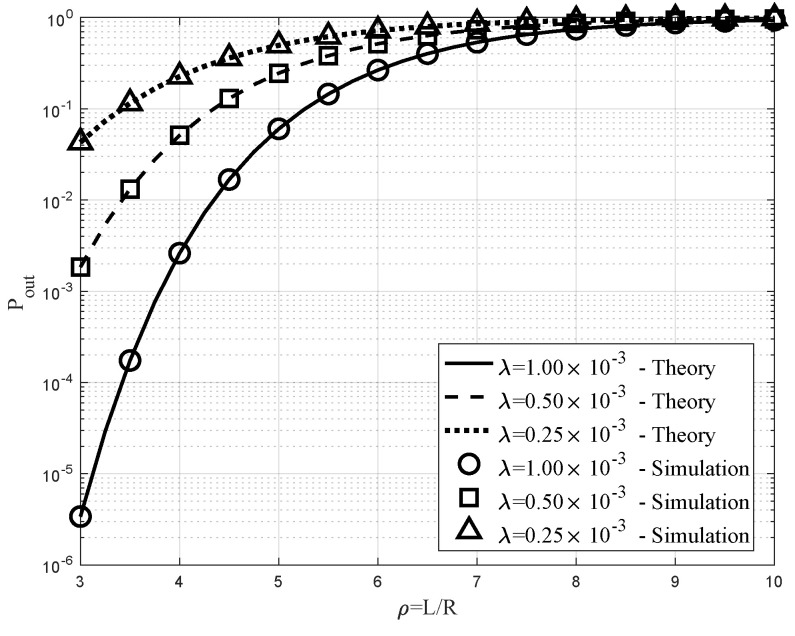
Outage rate versus ρ=L/R with $\beta =2.5\times 10^{-2}$, R=30 m, and λ={0.0025,0.0005,0.0010}.

Figure 7 shows similar outage rate versus ρ=L/R graphs but with different λ={0.0025,0.0005,0.0010}, which correspond to the solid, dashed, and dotted curves, respectively. We observed the same increasing behavior of $P_{out}$ as the ratio ρ increased. Furthermore, the simulation results were well matched with the theoretical results in this figure. In addition, comparing the three curves, it was observed that the lowest outage probability was achieved with the highest λ, since it could benefit from diversity gain in the presence of more UAV BSs.

### 6.4. $R_{opt}$ Verus ρ

**Figure 8:**
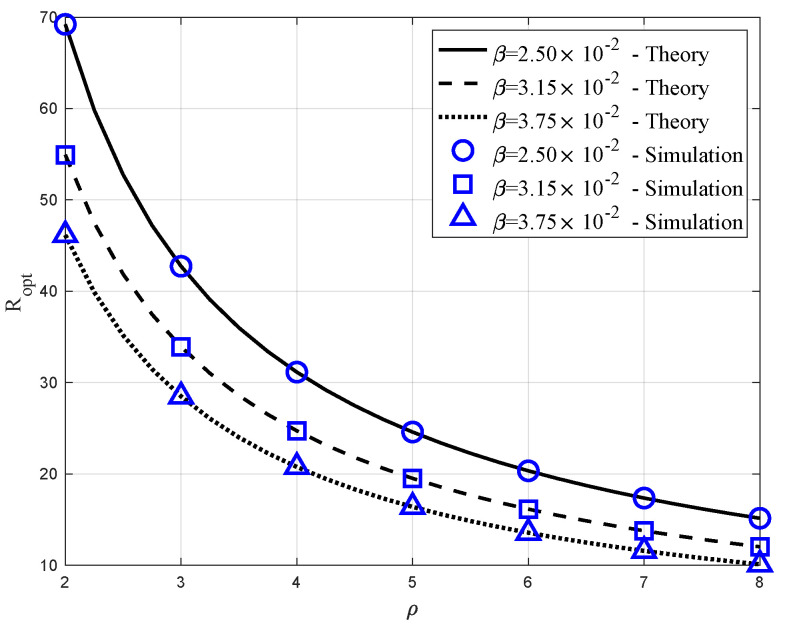
Optimal radius $R_{opt}$ versus ρ=L/R, when $\lambda =10^{-3}$ and $\beta =\{2.5\times 10^{-2},\;3.15\times 10^{-2},\;3.75\times 10^{-2}\}$.

Figure 8 depicts the optimal value of the radius $R_{opt}$ versus the ratio ρ=L/R graphs, which corresponds to the vertical and horizontal axes, respectively. We set $\lambda =10^{-3}$ and $\beta =\{2.5\times 10^{-2},\;3.15\times 10^{-2},\;3.75\times 10^{-2}\}$. The theoretical results with the three different β are represented by the solid, dashed, and dotted lines, while the corresponding simulation results are indicated by the three different markers. As found in Property 9, the optimal *R* decreased, as ρ increased. In addition, as in Property 8, $R_{opt}$ was an decreasing function of β, because higher β meant higher blockage probability for each link. We can also notice that the optimal value with $\lambda =10^{-3}$, $\beta =2.5\times 10^{-2}$, and ρ=5 in this figure is consistent with the result in Figure 5a.

## 7. Conclusions

In this paper, we consider a mmWave UAV networks, where multiple UAV BSs can provide connectivity to the distant user. Assuming uniformly distributed UAV BSs according to a homogeneous PPP, we first present the statistics of the distance between a typical UAV BS and the receiver, which determines the probability of the existence or absence of the LoS link. The approximate PDF of the distance, which gives the mean and variance in closed-form, is derived and validated by the empirical PDF obtained by random realizations. Using this approximate PDF, we derive the closed-form expression of the outage probability that no UAV BSs can connect to the receiver via LoS links. Further, we present nine properties of the outage rate under variations of different system parameters. In particular, we show there exists an optimal size (i.e., radius) of the spherical UAV swarm that minimizes the outage rate and derive the optimal value in a closed form. Through both simulation and numerical results, we observe that the outage rate increases as the blockage factor β increases. In contrast, the increase in the UAV BS density λ results in lower outage probability. In addition, we show that the optimal radius of the UAV swarm decreases with larger β and ρ=L/R.

This paper shows that it is highly effective to deploy multiple mmWave UAV BSs to provide better connectivity compared to terrestrial BSs with fixed locations, which suffer from random blockage effects. Furthermore, extended from the simple two-dimensional network, this paper investigates 3D distribution of UAV BSs, which benefits from higher spatial diversity to overcome the outage events of the conventional single-input-single-output (SISO) links. Future extensions of this work include other distributions of the UAV BSs (e.g., Gaussian and Laplace distributions) and cooperative beamforming with multiple UAVs to further enhance the coverage and capacity.

## Figures and Tables

**Figure 3:**
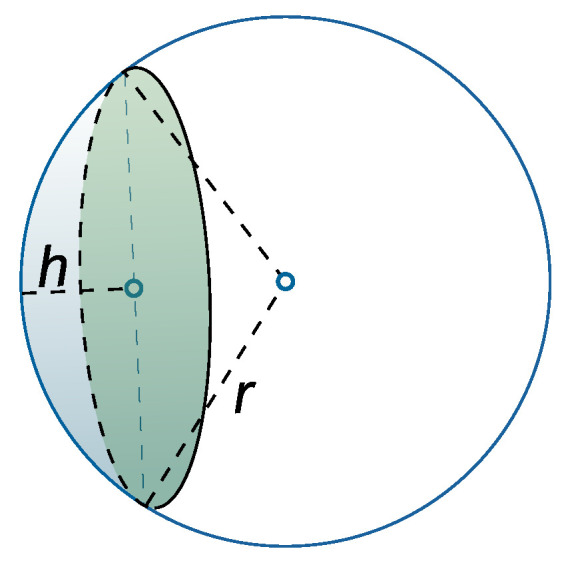
An example illustration of a spherical cap.

## Abbreviations

The following abbreviations are used in this manuscript:

5G	Fifth generation
A2G	Air-to-ground
AP	Access point
B5G	Beyond fifth generation
BS	Base station
CDF	Cumulative distribution function
LoS	Line-of-sight
mmWave	Millimeter-wave
PDF	Probability distribution function
PPP	Poisson point process
SISO	Single-input-single-output
UAV	Unmanned aerial vehicle
UDN	Ultra-dense network
UE	User equipment
